# Increasing person-centeredness in psychosis inpatient care: staff experiences from the Person-Centered Psychosis Care (PCPC) project

**DOI:** 10.1186/s12913-022-08008-z

**Published:** 2022-05-03

**Authors:** K. Allerby, A. Goulding, L. Ali, M. Waern

**Affiliations:** 1 Section of Psychiatry and Neurochemistry, Institute of Neuroscience and Physiology, Sahlgrenska Academy, University of Gothenburg, Blå Stråket 15, 41345 Gothenburg, Sweden; 2 Region Västra Götaland, Psychosis Department, Sahlgrenska University Hospital, 41345 Gothenburg, Sweden; 3 Institute of Health Care Sciences, Centre for Person-Centered Care, Sahlgrenska Academy, University of Gothenburg, Box 100, 40530 Gothenburg, Sweden; 4 Region Västra Götaland, Psychiatry Department, Sahlgrenska University Hospital, 41345 Gothenburg, Sweden

**Keywords:** Person-centered care, Inpatient care, Psychosis, Staff experiences, Intervention

## Abstract

**Background:**

Interventions to increase person-centeredness in hospital care for persons with psychotic illness are needed. Changing care delivery is however a complex venture, requiring staff to reconsider their mindsets and ways of working. A multidisciplinary educational intervention for hospital staff at four wards was launched to increase person-centeredness in the care of patients with schizophrenia and similar psychoses. This study aims to explore staff experiences of working to increase person-centeredness.

**Methods:**

A heterogenic sample of staff (*n* = 23) from all participating wards were recruited for six focus group interviews. Semi-structured questions covered staff perceptions of person-centered care and the process of increasing person-centeredness. Transcribed data was analyzed using thematic analysis according to Braun and Clarke.

**Results:**

Staff viewed person-centered care as an approach rather than a method. They described central aspects of person-centered care, such as recognizing the patient as a capable person who can participate in her/his care. Statements further showed how these core features were put into practice. Changes related to the intervention were presented in terms of evolving patient and staff roles, improved contact with patients, more flexible care routines, and a more positive ward climate. Neither psychotic symptoms nor involuntary status were considered barriers for person-centered care, but organizational factors beyond staff control seemed to impact on implementation.

**Conclusions:**

After implementation, participants displayed good understanding of the core concepts of person-centered care in both thinking and action. They attributed several improvements in the care milieu to an increased level of person-centeredness. Psychotic behavior and involuntary treatment did not present major barriers to person-centered care. Findings suggest person-centered care is feasible in the psychosis inpatient setting and could improve quality of care.

**Trial registration:**

The study is part of a larger study evaluating the intervention Person-Centered Psychosis Care (PCPC). It was registered retrospectively at clinicaltrials.gov, identifier NCT03182283.

## Background

Psychiatric inpatient care is intended to promote recovery from the most acute phase of mental illness. Among examples of good practices reports however depict a care environment far from ideal [1]. Patients treated for psychoses and other severe mental illnesses describe not being respected as human beings or being reduced to a disease [2–4]. They try to cope with a restrictive care environment that is at times unsafe and incomprehensible [2, 3, 5]. They find themselves in a passive patient role, subordinate to ward staff and psychiatrists [2, 3, 6]. Staff, on their part, depict an everyday situation where they try to provide good care in a suboptimal environment, where the patriarchal structure limits their possibilities to work proactively [6–8]. Instead, they must react to acute situations. All of this hinders positive staff-patient encounters. Although it is clear from patients’ accounts that positive interactions with staff improve the inpatient experiences [2, 5, 9], low degrees of patient/staff interaction have been reported over the last decades [10, 11]. Numerous research reports conclude that psychiatric inpatient settings need to develop more collaborative, interactive, flexible and supportive care to improve conditions and outcome for both patients and staff [7, 11–15].

Person-centered care (PCC) has been suggested as an approach to better meet the demands of a more efficient, qualitative and ethical care for complex and chronic diseases [16, 17]. The value-based concept lacks a clear definition and different ways of interpreting PCC have been described in terms of being holistic and addressing the person as unique; addressing difficulties in everyday life; considering the person as an expert of his/her life; or acknowledging the person behind the illness [18]. Within psychiatry, PCC has been conceptualized as being culture-dependent (reliant on attitudes and procedures reflected by staff and the organization), inter-personal, and empowering [19]. A recent review describes a core of PCC in terms of reoccurring principles; empathy, respect and engagement, relationship, communication and shared decision-making, holistic and individualized focus along with coordinated care [20]. These principles address the complexity, uniqueness, capacity and reciprocity of each patient, basing PCC in the philosophy of personhood [17]. While PCC shares several of these principles with other concepts, such as patient-centered care, the latter is suggested to focus on a functional life where PCC aims at a meaningful life [20]. This aim puts PCC in very close relation to the psychiatric concept recovery-oriented practice, which further promotes connectedness, hope and optimism, identity and empowerment [21] and incorporates a societal dimension in the ambition of restoring patients’ lost access to society as worthy citizens [22]. As PCC is a model of care in which patients and staff work towards recovery, and a recovery-oriented organization is needed to fully work in a person-centered way, the two are considered to be intertwined [23].

Staff are gatekeepers in the transformation of care towards a more person-centered practice. Development of new ways of thinking and working is essential to this end. Being at the center of this process, staff perceptions provide valuable information on the process of improving care. Within oncological inpatient care, staff report experiencing opportunities for increased patient participation and improved teamwork following the implementation of structured PCC procedures [24]. However, they also report limitations in the practice of PCC as some actions or routines remain patriarchal or task oriented. Staff have described supportive leadership, staff qualities and person-centered attitudes along with inter-professional cooperation as facilitators for person-centered care [25–28]. Barriers include a culture that remains biomedically- or task-oriented [26, 27, 29], diverse understandings of PCC [27, 30], and practical aspects like time constraints or patient characteristics such as impaired ability to communicate [26, 27, 29–31].

Relatively few PCC implementation studies are set in psychiatric services why experiences from staff are scarce. One US study showed that outpatient staff embraced PCC, but tensions arose when trying to practice it within traditional organizations [32]. A person-centered care model, the Tidal Model [33], was implemented in a psychiatric forensic unit from which staff reported a shift of focus from patient to person, a more equal distribution of power, relationships marked by empathy and respect, improved collaboration and improvements in both quality of and satisfaction with their own work [34]. The complexity of implementing PCC in psychiatric care was highlighted by psychiatric emergency unit staff who described making use of practical parts of the model but losing the underpinning philosophy due to knowledge gaps or the task-oriented and biomedical agenda dominating ward [35]. Shared decision-making (SDM) is an important ingredient in PCC [36]. A recent integrative review [37] captured staff perceptions of how SDM enhanced relationships between patient and staff, and how, in turn, a good relationship facilitated SDM. Although SDM is highly accepted by staff, poor insight into patients’ circumstances and needs is seen as a limitation for staff participation, as are time restraints [37].

The objective of this study is to explore psychosis inpatient staff’s understanding of PCC, and their experiences of working to increase person-centeredness, following an educational intervention and the implementation phase that ensued. The intervention, Person-centered psychosis care (PCPC), aimed at increasing the overall level of person-centeredness at four psychosis wards in a Swedish major city [38, 39]. The educational intervention was based on the Gothenburg model of PCC which was developed in non-psychiatric care [17, 40], and used a participatory design. Staff evolved their person-centred thinking through six educational course days which were spread out over 6 months, interspersed with practical testing of person-centred features such as attaining a patient’s narrative, working in partnership with the patient to form care plans and documenting the agreements. Through the creation and testing of practical projects, participants then worked to transform their ward practices. One third of all staff attended the educational days, and remaining staff were involved through knowledge translational activities and involvement in the practical projects.

The educational intervention and implementation are described in more detail in the study protocol [38] which presents the planned evaluation of PCPC in its entirety.

## Method

### Design

This is a qualitative study based on focus group interviews. Aiming to explore staff experiences of a complex process, the focus group interview was considered appropriate as it effectively collects data and allows participants to expand on their individual perceptions of the shared experience in discussion with the other participants [41, 42].

### Study setting

Four psychosis wards, delivering inpatient care for all patients with schizophrenia and similar psychoses in major Swedish city participated in the intervention. Wards are mainly staffed by nurses and nurse assistants. A psychiatrist, assisted by residents and interns, is in charge of medical assessments and formal decisions at each ward. The services of psychologists, social service counselors, physiotherapists and occupational therapists are available and integrated in the inpatient care.

### Participants, sampling and recruitment

A purposive sample regarding gender, profession, length of employment, ward affiliation and role was recruited. Further, we wanted representation of staff who took part in the educational intervention, as well as those who took part in translational activities only. In a first step, all staff and heads at the four wards were invited by email. Posters in staff offices also provided information about the study. In a second step, assistant managers on all wards helped recruit staff by re-inviting all co-workers, and finally researchers approached specific persons to fill voids in the sample (mainly ward affiliation and role). Sampling continued until heterogeneity was achieved. Up to 8 participants were invited to each interview, but the number of participants was limited by ward incidents and forgetting to attend. A total of 23 persons participated in the 6 focus group interviews, with 2-5 participants per group (Table 1). Data collection started 10 months after completion of the education (spring of 2017) and ended nearly a year later.

**Table 1:** Background variables for staff participating in focus group interviews (*n* = 23)

Participants	
	**Mean (min-max)**
Age^a^	41.8 (22–63)
Years employed in psychiatric services	9.2 (1–41)
Years employed at clinic	4.4 (.25–17)
	**Nr (%)**
Gender	
Female	13 (56.5)
Profession	
Registered nurse	7 (30.4)
Nurse assistant	13 (56.5)
Social worker	1 (4.3)
Psychiatrist	2 (8.7)
Manager position	2 (8.7)
Participated in PCPC educational intervention	11 (47.8)
Experience of any type of PCC training prior to intervention	6 (26.1)
Previous experience of working with PCC	3 (13)

^a^Data missing for seven participants, calculations based on the remaining sample

### Data collection

All participants were informed of study aims and procedures, the voluntary nature of participation and the possibility of withdrawing consent without explanation at any time. Consent forms and background data were collected before the interview. All interviews took place at the clinic’s administrative unit, nearby the wards so staff could easily join from their shift. Each interview started with the setting of social rules to address the importance of a friendly, non-judgmental atmosphere. A semi-structured interview guide covering different areas of the PCPC project guided the interview, including questions such as “What does person-centered care mean to you” and “Compare how you worked before and after the PCC intervention”. The guide was drafted by the researchers. Each interview involved two researchers, one leading the interview and one supporting: AG, AL or KA. Participants were to different degrees familiar with the researchers, see section Author Information. The researchers introduced themselves, their professions and their various roles as researchers on the PCPC project. Interviews lasted between 41 and 53 minutes. All were audio recorded and transcribed verbatim by a professional transcriber, after which the first author listened to the recording and proofed the transcriptions.

### Data analysis

A thematic analysis was chosen as it suits our purpose of exploring the patterns that emerged from staff accounts. A realist perspective was adopted, assuming a fairly straightforward link between reality, experiences and narrative where focus is to analyze accounts of these experiences, without addressing interpretations of sociocultural contexts and structures influencing personal accounts [43]. Following this view and the aim of the study, the thematic analysis was conducted at a semantic level, according to the well-established 5 step procedure described by Braun and Clarke [43]. The first author read and reread all transcripts (step 1, familiarizing) and made an initial coding (step 2, labelling meaning-bearing units). One interview was double coded with a co-author (AG), and only minor coding differences were detected. NVivo 12 was used in the process of grouping codes into themes (step 3) and reviewing them (step 4, going back to meaning-bearing units). Co-authors (AG, LA) reviewed the thematization and discussions on diverse interpretations guided the analysis towards the final result, ending the process with naming themes (step 5). Excerpts were chosen carefully in order to retain anonymity. Participants were assigned fictitious names.

## Results

Three themes and nine sub-themes were identified (Table 2), which describe the participants’ understanding and expectations of person-centered care along with its realization in practice, experiences of change, and perceived facilitators and barriers for increasing person-centered care.

**Table 2:** Themes and sub-themes representing staff members’ perceptions of working to increase person-centered care

Themes	Sub-themes
From theory to practice	The theoretical understanding of person-centered care
	Expectations on increasing person-centeredness
	Person-centeredness put into practice
Experiences of change	Improved relations
	Patient engagement
	Professional growth
	A better care environment
	Lack of change
Barriers and facilitators for person-centered care	Barriers
	Facilitators

### From theory to practice

This theme reflects how staff assimilated the two parts of the intervention and implementation; enhancing person-centeredness in their way of thinking, and changing clinical practice. Three sub-themes emerged that showed a theoretical understanding of the concept of PCC, staff members’ expectations linked to that understanding and how this was put into practice.

#### The theoretical understanding of person-centered care

Most participants understood person-centered care as an ethical care approach rather than as a method, exemplified by Erik: “There’s no recipe, no template to follow. It’s an approach”. The content of the approach was described as viewing the patient as a person, and involved seeing beyond symptoms, exploring who the person is, and what resources s/he possesses. Stefan: “Instead of thinking [s/he is a] patient, the person is the focus … a human being with own wishes, dreams … explanations … what one wants in life”.

Acknowledging the patient’s capacity seemed to be a central aspect in PCC. Participants talked about the importance of identifying the patients’ resources and utilizing them in the care process. “It’s to make the most of the patient’s … or person’s … resources and encourage that they are used” (Stefan). Resources included the patient’s network but could also be anything that affected health, including the patient’s personal qualities. Patient participation was highlighted as a core feature of PCC, as Sara said: “Above all else, the patient should be involved and participate in his/her care”. Some participants elaborated that participation is about giving the patient a say. Sara continued: “… be able to participate in their own care, [it’s about] what they want. Not [about what we] tell them should be done “. Participation included sharing responsibility with the patient and respecting her/his autonomy. Shared decision-making was considered an important part of person-centered care.

Individualized care emerged as another main feature of person-centered care. This was described in terms of tailoring care to fit the unique patient. Karin expressed it as: “...we see the individual and … what fits her/him”.

#### Expectations on increasing person-centeredness

The participants expected increased person centeredness as they worked towards that end, and with it, better quality of care. A recurring response was that they hoped that patients would become more actively involved in the care process, exemplified by Nils: “I hope patients will sit [with the team] and discuss their medication and health”.

Increased patient participation was in turn expected to reduce care consumption. Improved information and communication, along with individualizing care was thought to reduce length of hospital stay. Astrid summarized:” if you’re treated well, you simply feel better, and if you’re able to say what you need and get help with that, then the hospital stay should reasonably become shorter”. Carl added; “clear information is also enormously important … [for the patient to] be able to think … ‘maybe it’s a good idea for me to continue [taking the medication] … increases the chance of avoiding re-admittance “.

Enhanced patient involvement was also expected to generate a safer workplace for staff, which was exemplified by Andreas: “… a gain for staff … become less exposed to threats and violence … So many times [before] I’ve been in the firing line when distributing medicines because I thought the patient had been informed [of the medication change] when this wasn’t the case. I think that [increasing person centeredness] can make it easier for us”.

#### Person-centeredness put into practice

Practicing person-centered care emerged as a conscious and active choice of actions with the overall goal of including the patient in the care process. This is exemplified throughout the sub-theme both by how staff interacted with patients, and by the organizational changes they made to facilitate these interactions.

Staff-patient conversations emerged as a major point of person-centered actions. The participants described enhanced awareness of how they talk to each patient, adapting their communication to that person. Bosse stated: “...it’s in the back of your mind, during conversations and so on, your starting point is the person you’re talking to “.

Giving information and listening to patients’ narratives emerged as two important components of the conversation. Providing patients with the information they needed was thought of as an important feature in patient participation, and study participants described how they elaborate answers to patients’ questions, making the patients aware of what the inpatient stay will include such as mandatory and optional features, explaining why things are done, and being transparent about staff assessments. Ulrika summarized: “We, staff … talk to the patients … [so that] they are more informed compared to before”. Staff prioritized talk with patients over other tasks. This included listening to patient narratives which was explicitly mentioned as an important source of information, enhancing staff members’ understanding of, and co-operation with, the patient. A third aspect of conversation involved the patient and the staff member working together towards a shared goal. Through joint discussions they together decided the best way forward, which was summarized in the patient’s care plan.

The care plan emerged as one core feature of patient participation. Several participants related patients’ influence on care planning situations: “[The] patient gets an opportunity to express their own view of their problems, wishes, resources and treatment goals. We try to do [patient-staff care planning] more consistently … to get the patient’s own version of the situation even if it contradicts the doctor’s assessment “(Jenna). Patient participation in care planning was facilitated by helping patients to gather thoughts and questions, preparing them for the care planning meetings, writing the care plan together with the patient, and reviewing it afterwards: “We try to invite the patients to participate more in their care plan … go through what will be discussed [with the doctor] “(Kajsa).

Although rarely mentioned explicitly, shared decision-making in practice was exemplified. Matilda described how procedure for treatment decisions changed: “… we rarely decide for the patient [at rounds] except for involuntary procedures … instead we have a conversation with the patient“. In involuntary care situations the patient’s preferences were described to guide the procedure as far as possible, for example by letting the patient choose who is present during the administration of forced injections, or in which room the injection is administered. Jenna said: “[the patient can] choose if my colleague or I give the injection … you listen to the person’s wishes”. Allowing patient requests to steer staff actions also emerged in participants’ examples of everyday situations: “...a patient had a request and then we looked at the whole picture and thought yes, absolutely, we do it that way because that’s the best way” (Lena).

In order to facilitate these aspects of PCC, routines needed to change or to be used with greater flexibility. Participants described the start-up of regularly planned meetings for the patient with doctors or contact persons as one example of new routines to facilitate participation (a contact person is a member of staff assigned to a patient to work as an “inpatient case manager”). This allowed patients to plan their time and know that they get to see the doctor and the contact person on a regular basis. By allowing flexibility in rules and routines, staff were able to make individual adaptations, such as serving coffee during the night or compromising on times for temporary leave from the hospital to accommodate patients’ needs. Statements further show an ongoing reflection in staff in cases when a patient’s request or the good of that patient did not match with existing routines. Kajsa described:” ...it’s about what the patient needs and us not putting up obstacles to satisfy that, because even if there were strict rules before, ‘don’t do this, don’t do that’, [now] we always say why it’s not possible to carry out …. We have become even better at questioning ourselves before we say no“.

### Experiences of change

Staff described changes resulting from their improved person-centered approach and routines, and these clustered around contacts with the patients, personal or professional development, and the care environment. A final sub-theme summarizes the experience that nothing had changed as a result of the PCPC project.

#### Improved relations

Several statements reflect change in contact with patients, adding up to improved relations. Ida evaluated the PCPC project: “I think it has created a bit more contact with the patients, in a positive way“. Other participants expressed how the everyday conversations in the hallway such as a simple “how are you today” confirm the patients as persons. Tina described how listening to patients creates an atmosphere of security for the patient: “I have a patient now who feels very secure since I sit and listen when she tells her story. Even if it’s crazy, it’s her story”. These qualitative changes seemingly affected patients, as they turn to the ward for support also after discharge: “We get many more calls from former patients … [who say] ‘you’re the only ones listening to me’” (Patrick).

Participants described how giving clearer information decreased the need for repeated questions from patients, which gave space for other conversations. The way information was given was touched upon in descriptions of how participants more often address patients’ questions with kind explanations instead of just a “no”. Amina summarized how the relations with both patients and others involved changed with better communication and information: “[Patients] get a greater understanding of why certain things happened at the ward, [there are] fewer questions at discharge which has led to … more gratitude, especially from next-of-kin … It’s a big difference”.

Furthermore, improved relations with the patients’ network, both next-of-kin and the psychosis outpatient care clinic, emerged. In Sweden, psychiatric outpatient care is run by separate teams who, with varying success, collaborate with the inpatient setting when patients are admitted to hospital. Next-of-kin were more often invited to take part in the care process and kept more informed. Contacts with supported housing staff were described as more open and supportive, and co-operation with outpatient service staff was experienced as smoother by some.

#### Patient engagement

Participants depicted change that implied an evolvement of a more active patient role. Patients were perceived as participating more in the care process, as Kajsa stated: “If we did a patient survey now of patient involvement [in their care] ...or having knowledge of their care plans … I think this has increased significantly compared to before“. Participants described patients asking more relevant questions and demanding information. Anna exemplified this: “In my experience patients are more involved … both next-of-kin and patients ask for information more often, like ‘I should get this information’ “.

There were also statements depicting individual responses from patients, such as surprise when invited to participate, which implies patients noting changes. Other participants shared experiences of patients having an increased sense of self-worth, which they related to staff’s work to increase patient participation. There were also descriptions of patients being more secure, hopeful, happier and as having a more grateful attitude to staff, like Amina’s experience: “I’ve seen more gratitude and hope in patients”.

#### Professional growth

Professional roles were suggested to have developed as well. Assistant nurses felt that their work had become more important for the professional team as they were much involved in attaining the patient narrative and working with the care plan together with the patient. Erik exemplified expanded responsibilities: “You get to develop your profession … keeping the contact with outpatient care, network meetings, care planning … It’s great fun...“.

Participants also talked about how they had changed as persons in terms of increased reflection on the way they carry out their work. This was exemplified by Lena: “I have thought about what and how I say things … I’ve tried to really think about that...So that’s how I’ve been affected...“. Statements suggest that realizing how much patients want to participate in their care creates humility and a wish to include them even more. A greater interest in patients as individuals and a better approach to patients as a group was put forward, which seemingly made work more rewarding, as Kajsa described:” You gain such incredible knowledge of the patient through his/her narrative … it makes the work more fun and interesting”. Moreover, greater commitment to the job was reflected on, which was considered positive, although sometimes coming with a price in terms of exhaustion and disappointment. Amina described: “...I’m sometimes exhausted because I’ve given so much of myself, and then when all these doors close [opportunities for the patient], you stand there beside the patient … feeling just as stuck...So [engaging more] is both positive and negative.”

#### A better care environment

Experiences of an overall improved care environment emerged; both ward climate and work situation were described as improved.

The ward climate was described as more open, equal, and calm. Matilda stated: “nicer climate between staff and patients. It’s sort of more equal, [we’re] at the same level“. Kajsa added to this notion: “you notice a greater calm on the ward, and I think it has to do with patients being noticed and confirmed … more than before”. Other participants related the greater calm to reduced levels of frustration or worry in patients, exemplified by Patrick: “I actually experience the patients as calmer … not as much of this frustrated anger“. Participants related further that they felt more comfortable being around the patients, to let them “come close”.

Moreover, several participants noted a decrease in the use of involuntary procedures, which in turn was related to a decrease of threatening or violent situations. This was exemplified by Jenna: “we have come quite a long way in decreasing involuntary procedures...“. Statements link this change to staff being more tolerant with disturbing patient behaviors, trying to meet patients with dialogue, and the flexibility in routines allowing for example a restless patient to go for a smoke during the night. Amina explained: “… we’re much faster to read [a situation] … this [patient] is someone who provokes. It’s enough to say something and [the patient says] ‘whatever, it’s cool’. And that’s great. I think it’s because we have a generally more open climate now“.

Turning to the work situation, participants described a smoother job flow, with generally fewer interruptions. They related this to improved relations with patients, new routines, and improved information transfer. Matilda exemplified: “I have better flow at work. Or I experience less resistance … now when there’s a better climate with the patients“. The participants also described improvement regarding teamwork and emphasized that psychiatrists are more involved in the teamwork: “The doctors are more involved … we work more as a team, and we [staff] are allowed to have opinions … “(Sara).

#### Lack of change

Not all participants experienced change following the work to increase person-centeredness. There were reflections that no personal changes had occurred, such as Sverre: “So I think [focusing on person-centeredness] is fun, but I can’t really say that it affected me in any way“. The cooperation with outpatient services was unaffected according to some participants. One participant expressed seeing no noticeable changes following the PCPC intervention, remarking that person-centeredness was new in name only; it had already been around for a long time.

### Barriers and facilitators for person-centered care

Participants addressed both direct and indirect obstacles when working to increase PCC, but also highlighted several facilitators.

#### Barriers

The barriers discussed were related to both theoretical and practical aspects. A theoretical aspect concerned the complexity and “fuzziness” of person-centered care as a concept, which participants meant complicated their understanding. Matilda exemplified: “For me it’s been a disadvantage not to be able to define it properly. It [how to work according to person-centered care principles] can’t be written in a routine document.”. Developing an understanding of what person-centered care is and how to put it into practice required time and effort. Participants said that it was difficult to convey the concept of person-centered care to colleagues. For example, Kamal stated: “… [when colleagues asked] ‘what is PCPC?’ [I answered] ‘I don’t know’ … it’s a way of thinking, and to try to explain a way of thinking … it’s almost impossible“. Two years into the implementation process, participants still found it difficult to fully understand what person-centered care is. Jenna described the situation of a new resident: “She had never encountered this before and suddenly she’s expected to do this and do that and understand the concept that we after two years still have difficulties getting a grip on“. Diverse interpretations of PCC emerged in the process of translating PCC into practice. Statements show how staff struggled to find common ground, exemplified by Kamal as: “It’s always easy to agree on routines and boundaries … but when it comes to this more personal touch … what’s ‘crossing the line’ for me is maybe not ‘crossing the line’ for you …”.

The everyday work situation at the ward was described as a barrier by both management and staff. Time is limited and filled with tasks, leaving little room for work on care improvement. Kajsa concluded: “But it [a practical project that was planned as part of the PCC intervention] didn’t happen because we’re so busy with everyday work”. Statements addressing the need for additional resources confirmed this obstacle. Staff turnover was also put forward as a barrier pertaining to the everyday situation, as it takes time and effort to recruit and introduce new colleagues.

Interplay between staff members was also reported as an arena for barriers. Diverse opinions and differences in levels of engagement were described as hampering implementation: “I find it problematic that not everyone has been on board. We have staff, management, regardless of profession … who expressed ‘I don’t want to work like that’. So, if some want to work like that, and some don’t, it gets problematic …” (Amina). Sub-optimal co-operation between psychiatrists and care staff was explicitly mentioned as a problem; new person-centered procedures were at times disregarded by psychiatrists. There were also some more implicitly expressed concerns that new strategies and routines were not adopted by all, thereby reducing the effect of the intervention. Jenna said: “...since it’s been a bit messy among us [staff] on what this [person-centered psychosis care] should become, and everyone hasn’t been up to date with the new routines, I’m afraid the patients feel it’s messy … that we don’t really listen anyway“.

A final area of obstacles relates to the interplay between patients and staff. One potential barrier revolved around staff’s ability to engage in qualitative conversations with patients, as Carl explained: “But it’s difficult … talking about their symptoms … their thoughts about [symptoms] and whether they have a disorder … what the doctor says. It’s really difficult, not.

something that’s easy to learn“. Other participants discussed the heterogeneity in patients’ culture and language as a potential difficulty, especially the use of interpreters which was considered to have a negative effect on communication with patients. A final obstacle was that as person-centered care allows for different routines or rules for different patients, staff.

must handle” inequality” as patients notice that not everyone has the same privileges, for example to use the outdoor area, or the phone. Amina explained: “… discussions arise [with patients] … but I sometimes feel it’s more difficult not becoming too personal regarding information [when explaining to a patient that] ‘this person gets to use our phone because … ’ I shouldn’t have to tell one patient about another … it can be tricky sometimes“.

#### Facilitators

Participants expressed how various resources and strategies were helpful to increase PCC. Many of them were staff related. Statements bring about members who functioned as informal enthusiasts; they were seen to drive the project forward. There was also a formally designated “resource person”, a staff member assigned to assist PCC implementation on all four wards. Participants found this very valuable. For example, Sverre said: “I see the difference now that [the resource person] has left his calendar for co-workers to book appointments when needing help or support to talk to patients and write care plans. I notice [this work] gets done more often. Several [co-workers who] didn’t dare to do it, now do it with help from [the resource person]“. Participants also mentioned support from medical students who were tasked to monitor care plans during the summer holiday period. This ensured that new routines regarding care planning were not lost during the summer when many staff were temporary summer substitutes untrained in person-centered care. Another type of resource, the steering group, was mentioned by ward managers. The steering group included the head of the Psychosis Department, researchers, and managers from each of the four wards. Ward managers found support in the group which met on a regular basis to discuss progress, to troubleshoot problems, and to find solutions. Kajsa said: “We had these follow-up meetings … been a driving force these years since we left [the educational days] … it’s been very helpful”. On a more general level, participants regarded their individual differences as strengths, bringing diverse perspectives on a situation, and opening for new resolutions. Some remarked that a solution-focused attitude was particularly helpful. Jenna summarized the effect of such a mindset: “Yes, I think it gets better and better...because we’ve tried to find solutions”. New or younger co-workers were seen as an asset; they could quickly pick up the person-centered care approach. Furthermore, some recently graduated staff members had already been introduced to a person-centered approach during their nursing education.

Specific components of PCC were identified as facilitators for change. Participants explicitly mentioned working with person-centered care plans as a facilitator per se. This new routine united staff members, providing an important focus for implementation work. Another component was the person-centered communication style. Several statements reflect how staff engage in discussion to advance their understanding and to solve disagreements regarding care practices. This was considered a powerful tool in further advancing person-centered care. Patrick stated: “Now we have an open discussion on everything and then there’s no problem”.

## Discussion

Participants understood person-centered care as being about patient involvement in care and care being tailored to fit the individual. This understanding was translated into practice, described by participants as deliberate work to activate the patients, providing information and preparing patients for shared decision-making during care planning. The view of the patient as a capable person was seen as the underlying assumption in support of patient participation. This was also expressed more directly by staff in terms of identifying an individual patient’s skills and resources and encouraging their involvement both during and after the ward stay. PCC was perceived as an approach, rather than a method. This contrasts with findings of a previous study [35], in which staff adopted certain methods but not the philosophy of PCC. Staff experiences in our study might in part be explained by the character of the intervention which emphasized patient-as-person as the foundation, and encouraged individualized ways to acknowledge this in practice [38], rather than providing a preset structure that staff were expected to follow [44].

Several participants addressed individualized care as a core feature which is related to the view of the patient as a unique person. A willingness to understand this person is illustrated by statements highlighting the power of the narrative. Statements suggested a patient’s choices might become understandable, if she is allowed to tell her story; she is not rejected as crazy or uncooperative. On one hand this represents person-centeredness as the patient is given credibility and acknowledged as a part with a say; on the other hand, it reflects an ongoing interpretative precedence as staff overlay their interpretation of the patient’s story. We believe this is an important area of discussion, as it involves a potential conflict in trusting a patient’s capacity while s/he is affected by psychotic symptoms. Previous researchers have concluded that inpatients with schizophrenia are able to participate in shared decision-making processes [45]. On an individual level they must however face epistemic injustice, the discrimination, based on prejudice, of their capacity as a knower, implicated by both the patient role [46] and the psychosis itself [47]. For the patient’s narrative to become the opening door to a truly person-centered care, staff need to reflect on how they assess and interpret the narrative.

Staff reported several changes related to the PCC intervention, one being improved relations. Improved empathy, respect and contact have been reported from several settings after the implementation of PCC approaches [48], including inpatient forensic psychiatry [34]. Improved relations could be considered a key to other types of change. In our study several aspects of the improved care environment, such as job flow and satisfaction, related directly to patient relations. Improvements in job quality and satisfaction have been reported by staff in PCC studies set in other care settings [34, 49]. Enhancing patient relations might be a means of improving staff work experience. On the other hand, our findings suggest tensions between improved job satisfaction and the emotional toll of engagement, previously described in non-psychiatric care settings [48]. This has also been expressed in terms of stress as staff have to try to fit in features of PCC into traditional care delivery models or balancing conflicting priorities [26, 44]. Our findings, taken together with those reports suggest that not only staff but also the care organization itself must align with PCC.

Participants described change in patient behavior; patients were more active and, in some cases, demanded more of staff. This was suggested to be a direct effect of patient inclusion in the work with care plans, as well as the improved sharing of information. Similar experiences were reported from medical wards after the implementation of PCC; patients who were well-informed were aware of their capability and influence, which seems to have increased their satisfaction and engagement in self-care [50]. Our study also suggest a greater calm in patients, related to improved structure and communication which aligns with previous findings of patients being more calm and independent with PCC in place, while losing self-esteem and becoming passive in non-PCC practice [48]. Results suggest that if patients are given the opportunity and knowledge, their role in the care process can evolve, which reflects the very basis of PCC; the philosophy of the person as someone capable.

Several barriers for the implementation and practice of PCC emerged in our study. The complexity of the concept of PCC was put forward and participants depicted divergent understandings of the abstract concept, which became apparent when staff endeavored to translate theory into practice. This issue has been reported by staff and researchers from a wide range of health care settings, including psychiatric inpatient care [27, 48]. When implementing PCC as a practical routine, there is a risk that the essence of the approach -to acknowledge the personhood of the patient- can be lost. Participants also described differences in the degree of staff engagement, relating this to variation in both understanding and interest, an issue previously reported in several PCC implementation studies [48, 51]. Participants directly addressed a gap between doctors and the rest of staff. This has been addressed by PCC-researchers who attributed the power relations built around doctors as a barrier for change [26]. Although the traditional hierarchy of the care organization runs deep, PCC might influence power relations on the ward. In our study nursing assistants describe an evolving role with more responsibility and credibility in the care team, and nurses described improved teamwork, suggesting the dynamics of the person-centered approach improved collaboration within the care team.

Lack of time and everyday tasks taking precedence over PCC innovations were also put forward as barriers for change in our study. Similar findings are reported from other PCC implementations [27, 51]. Parallel to this finding, reports suggest that once PCC is implemented it actually saves time, as patients goals are more efficiently supported and patients themselves take more responsibility [26]. There are also reports of staff slipping back to task-oriented care when the workload becomes high [49], suggesting that successful implementation of PCC needs to overcome both the initial struggle while working to establish change, as well as the more long term situation since burdensome ward situations might trigger old behaviors. Managers in this and other studies [25] addressed the disruptive nature of staff turn-over, acknowledging the importance of continuous work within the care model. The current situation with high staff turnover and recruitment difficulties is a major challenge. PCC might however be a way to retain, and attract, staff. Improved ward climate is suggested in this study, and studies within outpatient psychiatry have suggested that the culture and climate of the organization impact on staff attitudes, which in turn predict turnover [52]. Further, one participant in our study described actively seeking employment at our clinic because of our ongoing work with PCC, others talked of improved work situation in terms of more fun, engaging and smooth job. Quantitative evaluations of work attitudes such as job satisfaction are however needed in the inpatient setting as positive associations between PCC and job satisfaction reported from geriatric care settings [53–56] cannot be directly extrapolated.

As acute psychosis severely affects cognitive processes and social interactions, we had anticipated that participants would highlight illness-related issues as barriers for PCC implementation. Such reports were uncommon, and the same was the case for involuntary care procedures. Coercive procedures were not seen as barriers, but rather situations in which the PCC approach was considered particularly relevant. Earlier studies are in line with this thinking, finding PCC possible to practice even within involuntary situations [57]. An issue that surfaced regarding patient interaction was instead related to language difficulties. The use of interpreters hampered the patient-staff connection, as noted in other care settings [26, 31]. This was anticipated as conversation is the natural starting point for a person-centered relation.

Participants in our study identified both informal and formal facilitators of PCC. The former included a solution-oriented mindset as well as the heterogeneity of the team which stimulated new approaches to care. Formal facilitators were medical students (summer only) and a resource person (year-round) who aided and stimulated their co-workers in increasing person-centeredness in everyday care tasks. The fact that the resource person was much appreciated by staff, as well as participants’ explicit wish for continuous and structured supervision demonstrates the need for long-term support for the development of person-centered care approaches over time. Such a need has been suggested in the dementia care setting [28], and seems highly relevant also in the care of persons with serious mental illness.

The results of this study provide a deeper understanding of PCC in a hospital setting for persons with acute psychotic illness. The qualitative approach and the non-randomized sample of participants however makes the transferability of results to other settings limited and caution is warranted when making conclusions for the study population. The small sample size might limit the range of staff experiences and thus the result. However, all ward based professions and roles were represented (including psychiatrists). The professions and roles not represented (psychologist, physiotherapist, occupational therapist) were those that are called in as consultants when needed. Recruiting was purposive, to ensure a wide range of experiences in the data. Participants were recruited partially by open invitation, which might lead to an overrepresentation of staff who were positive to the intervention. Our results reflect staff members’ subjective experiences, and outcomes measured in an objective manner might differ. Previous research has shown that the presence of a person-centered thinking doesn’t necessarily result in person-centered actions [58], and care staff tend to overrate their performance [59]. While our study provides snapshots depicting actual delivery of PCC, we do not know the extent to which patients actually received person-centered care. A study limitation is the lack of an objective measure of person-centeredness at the ward level, which could allow for triangulation of our findings.

We chose to collect data through focus group interviews which comes with some considerations related to the interaction of participants. Participants might be affected by each other, conforming to the group’s overall thoughts or withholding statements that might be unwelcomed. Interviewers tried to access the divergent perspectives of all participants, and both “positive” and “negative” statements were encouraged. Still, interviews with individual staff members might have yielded other results.

## Conclusions

Following the educational intervention and implementation, aiming at increased person-centered care, participants displayed a good understanding of the core principles of PCC and knew how to put these ideas into action in inpatient care for persons with psychotic disorders. Improvements in the care milieu were described and attributed to the increased level of PCC. Features specific to psychotic behavior or involuntary treatment did not present major barriers for PCC. This suggest PCC is feasible for the inpatient setting for persons with psychotic disorder, and could provide needed enhancement of care quality.

## Data Availability

The datasets generated and analysed during the current study are not publicly available as this has not been approved by the Ethics Board or communicated in study information to participants, but data are available from the corresponding author on reasonable request.

## Abbreviations

PCC: Person-Centered Care
PCPC: Person-centered Psychosis Care
